# Predictive accuracy of surgeon gestalt for adverse postoperative outcomes: systematic review

**DOI:** 10.1093/bjs/znaf249

**Published:** 2025-11-29

**Authors:** Jun J Yang, Samuel J Mullan, Tahmid M Rayhan, Ciaran K S S Sandhu, Anshu N Ramaiya, Carl J Heneghan

**Affiliations:** School of Medicine and Biomedical Sciences, University of Oxford, John Radcliffe Hospital, Oxford, UK; School of Medicine and Biomedical Sciences, University of Oxford, John Radcliffe Hospital, Oxford, UK; School of Medicine and Biomedical Sciences, University of Oxford, John Radcliffe Hospital, Oxford, UK; School of Medicine and Biomedical Sciences, University of Oxford, John Radcliffe Hospital, Oxford, UK; School of Medicine and Biomedical Sciences, University of Oxford, John Radcliffe Hospital, Oxford, UK; Nuffield Department of Primary Healthcare Sciences, University of Oxford, Oxford, UK; Centre for Evidence-Based Medicine, University of Oxford, Oxford, UK

## Abstract

**Background:**

Risk assessment plays an important role in surgical decision-making. To estimate complication risk, many surgeons rely on gestalt, a mental process that involves integrating a range of clinical information. Others utilize dedicated risk scoring tools, which offer more standardized assessments. The aims of this systematic review were to explore the current evidence on the predictive value of gestalt for adverse postoperative events and to compare gestalt prediction with various scoring tools.

**Methods:**

This systematic review was conducted following the PRISMA 2020 guidelines and the *Cochrane Handbook for Systematic Reviews of Diagnostic Test Accuracy*. MEDLINE, Embase, Scopus, ClinicalTrials.gov, ACM digital library, and IEEE Xplore databases were searched. Studies concerned with surgeon gestalt prediction of adverse postoperative outcomes were included. Risk of bias was assessed using the QUADAS-2 tool. Outcomes evaluated were gestalt and scoring tool predictive accuracies for mortality and morbidity. A narrative synthesis was conducted.

**Results:**

A total of 34 studies encompassing 33 657 patients were included. Surgeons had good discrimination when predicting mortality, but consistently overestimated risk. Scoring tools generally outperformed surgeons, but integrated tools incorporating both gestalt and scoring tool outputs performed best. There was some evidence that gestalt accuracy improved with surgeon experience. Surgeons may also be better at predicting complications for elective procedures compared with emergency procedures.

**Conclusion:**

Surgeon gestalt can be a valuable predictor of surgical outcomes both on its own and as a component of integrated risk scoring tools. Future studies should aim to elucidate what factors contribute to effective gestalt assessment.

## Teaser

In this systematic review, the predictive accuracy of surgeon gestalt for adverse postoperative events is explored. Surgeons consistently overestimate mortality risk, but have good overall predictive accuracy. Surgeon intuition is generally outperformed by systematic scoring tools.

## Introduction

Surgery can be dangerous. Approximately 3% of patients die within 30 days of surgery and around 14% develop postoperative complications^1–3^. Predicting the most likely postoperative outcomes, particularly adverse events, is important to inform clinical decision-making. These risk assessments allow for cost–benefit analysis when deciding whether a patient should have surgery; over-optimistic predictions can expose patients to excess risk, whilst pessimistic projections may deny patients the benefits of surgery^4^. Additionally, accurate outcome predictions are needed to inform patient consent^5^. They also aid management, allowing appropriate preparations to be made for the most likely complications^6^. Furthermore, outcome prediction is a useful component of quality assurance schemes; stratifying patients by risk allows investigators to account for severity of illness when comparing outcomes within or across surgical centres^7^.

Predicting postoperative outcomes involves collecting different pieces of information and assigning them weights according to their perceived importance. A bias may then be applied to positively or negatively skew the final prediction^8^. Surgeons often make predictions using ‘gestalt’, meaning this process of integrating information is done mentally, as opposed to with an external aid. The process may be conscious and logical, or subconscious and experienced as a ‘gut feeling’^9^. As well as considering tangible clinical data such as patient age, co-morbidities, and procedure invasiveness, gestalt may incorporate unmeasurable information such as an ‘end of bed’ impression or surgeon confidence in the theatre team. Different surgeons may choose to consider different pieces of information, weigh them differently, and assign a different bias, depending on mood, personality, and experience^10^. Consequently, relying on gestalt assessment alone can result in significant heterogeneity^11^.

To increase standardization of risk assessment, numerous surgical scoring tools have been introduced that generate predictions based on fixed variables with predetermined weightings^12^. These variables are often chosen using a combination of logistic regression models and expert opinion^13^. Recently, tools have also been developed using machine learning, which chooses variables and weightings without explicit programming after ‘learning’ from clinical data^14–16^. The scope of scoring tools ranges from general-purpose calculators (that may be applied to almost any operation and can predict many different adverse events) to specialized measures designed for use in particular procedures or to predict certain complications^17–19^. However, although scoring tools offer more standardized assessments than gestalt, they are not universally used owing to varying accessibility, poor integration into digital records, and some surgeons deeming them unnecessary^20^. Furthermore, despite the myriad available scoring tools, it is unclear if they are consistently superior to surgeon gestalt^21^.

The predictive accuracy of gestalt assessment was previously investigated in a 2019 systematic review^22^. However, new relevant studies have been published and there has been growing interest in using artificial intelligence (AI) for clinical decision-making. The aims of this systematic review were to provide an updated perspective on the predictive value of surgeon gestalt assessment for adverse postoperative events, to examine how accuracy is affected by factors such as surgeon training, to compare gestalt with established risk scoring tools, and to explore the clinical applicability of integrating gestalt into novel prediction models.

## Methods

This systematic review was conducted following the PRISMA 2020 guidelines and the *Cochrane Handbook for Systematic Reviews of Diagnostic Test Accuracy*^23,24^. The PRISMA 2020 checklist is shown in *Table S1*. The study protocol was prospectively registered on the Open Science Framework (OSF.IO/VYT3H). Due to extensive heterogeneity, initial plans for a meta-analysis were discarded in favour of a narrative synthesis, which was deemed more appropriate.

### Search strategy

J.J.Y. searched MEDLINE, Embase, Scopus, ClinicalTrials.gov, ACM digital library, and IEEE Xplore databases on 28 March 2024. ACM digital library and IEEE Xplore were included to find AI-related papers. S.J.M. extended the search on 20 June 2025 and records indexed after the first search were screened and extracted as described below. An ascendancy approach was used to search the reference lists of included papers. The search strategy, shown in *Table S2*, was designed in collaboration with an information specialist.

### Study screening

**Fig. 1:**
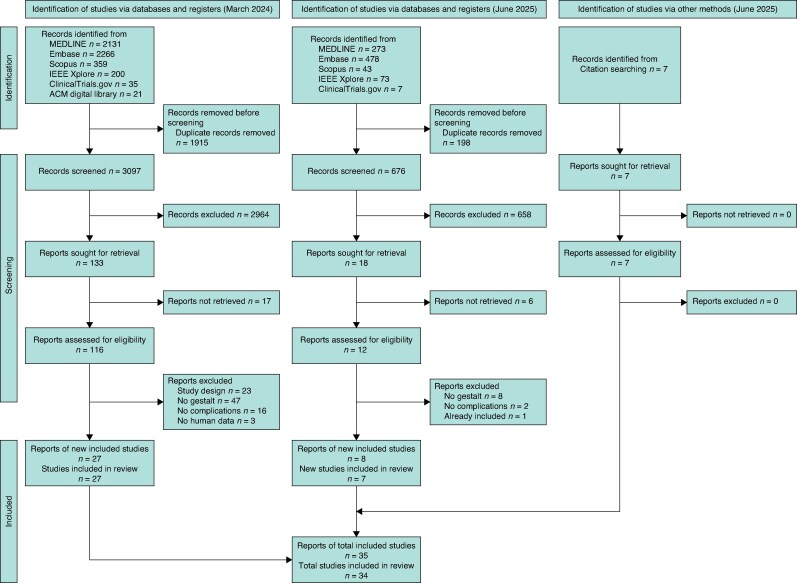
PRISMA flow diagram showing study identification and screening

Two pairs of reviewers (J.J.Y. and S.J.M., and T.M.R. and C.K.S.S.S.) screened identified studies’ titles and abstracts in duplicate against the inclusion criteria on Rayyan^25^. Included studies’ full texts were then screened in duplicate. Disputes were resolved by discussion and involvement of the other reviewer pair. *Figure 1* shows the PRISMA flow diagram^24^.

To be included, studies had to meet the following criteria: investigate predictive accuracy of surgeon gestalt for adverse postoperative events; involve one or more of the ten surgical specialties as outlined by the Royal College of Surgeons (cardiothoracic, general, neuro, oral and maxillofacial, otorhinolaryngology (ear/nose/throat (ENT)), paediatric, plastic, trauma and orthopaedic, urological, and vascular); and be a prospective cohort study, RCT, or retrospective cohort study.

Studies with the following characteristics were excluded: unavailable in English; involving non-surgical or simulated ‘hypothetical’ patients; no surgeon predictions made without decision aid (for example scoring tool); surgeons knew actual historical outcome before making their prediction; no full text was available; and no human data.

### Data extraction

Data were extracted using a predefined electric form (Google Sheets). Two pairs of reviewers (J.J.Y. and T.M.R., and C.K.S.S.S. and A.N.R.) extracted data from each study in duplicate. Disputes were resolved by discussion and involvement of the other reviewer pair. Where data was unavailable for extraction, study authors were contacted by e-mail requesting unpublished data. Where no response was received after the second e-mail, the study was excluded. A complete list of extraction items is shown in *Table S3*.

### Data analysis and statistical methods

The primary outcome of this review was the predictive accuracy of surgeon gestalt for postoperative mortality. Secondary outcomes were the predictive accuracy of surgeon gestalt for postoperative total morbidity and specific complications. Gestalt accuracy was also compared with various scoring tools, between surgeons of different seniority, and for elective *versus* emergency operations. The predictive accuracy was quantified by analysing discrimination and calibration where available. Discrimination was defined as the ability to accurately distinguish high risk and low risk patients by assigning them comparatively higher and lower risk predictions. This was assessed using the C-statistic from the receiver operating characteristic (ROC) curve. The thresholds for ‘acceptable’, ‘excellent’, and ‘outstanding’ discrimination were defined as 0.7, 0.8, and 0.9 respectively^26^. Calibration was defined as how well predictions aligned with actual outcomes. Calibration was assessed using the mean calibration. Mean calibration was calculated by dividing the mean prediction by the true rate^27,28^. Mean calibration scores >1 constitute an overestimation of risk; conversely, scores <1 constitute an underestimation. Potential causes of heterogeneity were explored through subgroup analysis: adverse event type (mortality *versus* morbidity); surgeon experience (junior *versus* senior); and procedure type (elective *versus* emergency). Graphs were produced using GraphPad PRISM 10.4.1.

### Risk of bias

Quality assessment for all studies was conducted using the QUADAS-2 tool for evaluating risk of bias and applicability of primary diagnostic accuracy studies in systematic reviews^29^. Two pairs of reviewers (J.J.Y. and T.M.R., and C.K.S.S.S. and A.N.R.) extracted data from each study in duplicate. Disputes were resolved by discussion and involvement of the other reviewer pair. For each study, risk of bias and applicability were each rated as low, moderate, or high, as shown in *Table S4*.

## Results

### Study characteristics

A total of 35 reports were included, encompassing data from 34 studies and 33 657 patients (see *Table 1*). One study was described in two separate reports, each considering different time frames (Gwilym 2022, Gwilym 2024). The primary outcome, surgeon gestalt predictive accuracy for mortality, was measured in 13 studies. The secondary outcome of surgeon gestalt predictive accuracy for morbidity was measured in 24 studies. A total of 30 studies involved in-person risk assessment of patients with subsequent follow-up to record the incidence of adverse events. Four studies were based on vignette-informed assessment, whereby surgeons were given data from previously completed surgical cases then asked to guess whether any complication had taken place during follow-up. A total of 28 studies compared surgeon gestalt predictive accuracy with some form of systematic scoring tool. Further study characteristics are shown in *Table 1*. Due to extensive heterogeneity, statistical testing was not done.

**Table 1:** Study characteristics

Study	Country	Number of centres	Specialty*	Number of surgeons	Number of patients	Follow-up	Assessment type	Assessment timing	Reference tool
**In-person risk assessment**									
Pettigrew 1986^SR1^	New Zealand	1	2	NR	218	Until discharge	VAS	Preop	None
Pettigrew 1987^SR2^	UK	1	2	NR	103	Until discharge	VAS	Preop, postop	None
Arvidsson 1996^SR3^	Sweden	1	1–8	NR	1361	Until discharge	VAS	Preop	None
Pons 1999^SR4^	Spain	7	1	NR	1198	30 days	Graded	Preop	Novel model
Avidan 2004^SR5^	UK	1	1	NR	210	NR	Binary	NR	None
Markus 2005^SR6^	Germany	1	2	5	1077	NR	Percentage	Postop	POSSUM, P-POSSUM
Kaafarani 2005^SR7^	USA	NR	2	NR	1621	2 years	Level of frustration	Postop	None
Hobson 2007^SR8^	UK	1	2, 3, 6	NR	163	60 days	Percentage	Preop	POSSUM, P-POSSUM
Woodfield 2007^SR9^	New Zealand	NR	2, 3, 6	58	1013	30 days	VAS	Preop, postop	Otago Audit Model
Smith 2008^SR10^	USA	1	2, 3	NR	57	Until discharge	Life expectancy	Preop	MSAS score
Burgos 2008^SR11^	Spain	1	5	3	232	90 days	VAS	NR	POSSUM, Goldman, Charlson
Karliczek 2009^SR12^	Netherlands	1	2	32	191	3 months	VAS	Postop	Novel model
Bakaeen 2010^SR13^	USA	44	1	NR	317	30 days	Percentage	Preop	CICSP
Cornwell 2012^SR14^	USA	44	1	8	181	180 days	Percentage	Preop	CICSP
Jain 2014^SR15^	USA	1	1	NR	5099	5 years	Percentage	Preop	VA CICSP
Glasgow 2014^SR16^	USA	2	2	*nr*	1791	30 days	Graded, percentage	Preop	NSQIP
Farges 2014^SR17^	France	9	2	3	946	90 days	VAS	Preop, postop	Novel model
Promberger 2014^SR18^	Austria	1	4	14	2558	Up to 12 months	Graded	Postop	Novel model
Ulyett 2015^SR19^	UK	1	2	NR	405	2.3 years	Rate of referral	Preop	Charlson
Sammour 2017^SR20^	Australia	1	2	NR	83	60 days	Percentage	Postop	Anastomotic leak risk calculator
Woodfield 2017^SR21^	UK	1	2, 6	58	859	30 days	VAS	Preop, postop	POSSUM, P-POSSUM
Samim 2018^SR22^	UK and Netherlands	3	2	12	349	30 days	Graded	Preop	Multiple†
Kohler 2018^SR23^	USA	1	5	3	270	30 days	Graded, percentage	Preop	None
George 2020^SR24^	USA	1008	6	NR	11 647	NR	Fitness for surgery	Preop	VQI-RAI
Vanbrugghe 2020^SR25^	France	2	2	5	101	7 days	Operation difficulty	Preop, postop	a-FRS
Zaruta 2023^SR26^	USA	1	5	1	98	12 months	VAS	Preop	None
Marwaha 2023^SR27^	USA	1	2, 4	216	216	30 days	Graded	Preop	NSQIP
Wu 2023^SR28^	China	1	1, 6	NR	161	30 days	Graded	Preop	GERAADA
Gwilym 2022/2024^SR29/SR30^	UK, Greece, Italy, New Zealand, USA Australia, Bahrain, Belgium, Germany, Poland, Portugal	38	6	567	537	12 months	Percentage	Preop	Multiple†
Berrigan 2025^SR31^	USA	1	2, 6, 8	11	286	30 days	Graded	Preop	Novel model
**Vignette risk assessment**									
Kumar 2017^SR32^	Canada	1	3	9	104	30 days	Operation difficulty	Preop	RENAL, PADUA, C-index
Dyas 2022^SR33^	USA	1	1	12	30	30 days	Percentage	Preop	SURPAS
Ishikita 2024^SR34^	Canada	1	1	1	25	5 years	Graded	Postop	AiTOR
El Moheb 2023^SR35^	USA	1	2	4	150	30 days	Percentage	Preop	POTTER

^*^1, cardiothoracic; 2, general; 3, urological; 4, otorhinolaryngology (ear/nose/throat (ENT)); 5, trauma and orthopaedic; 6, vascular; 7, neuro; 8, plastic. †Too many scoring tools used to be listed in main table; see *Table S5* for details. SR, Supplementary Reference (see *Table S7*); NR, not reported; VAS, Visual Analogue Scale; POSSUM, Physiological and Operative Severity Score for the Enumeration of Mortality and Morbidity; P-POSSUM, Portsmouth Physiological and Operative Severity Score for the Enumeration of Mortality and Morbidity; MSAS, Memorial Symptom Assessment Scale; CICSP, Continuous Improvement in Cardiac Surgery Program; VA, Veterans Affairs; NSQIP, National Surgical Quality Improvement Program; VQI-RAI, Vascular Quality Initiative Risk Assessment Index; a-FRS, alternative Fistula Risk Score; GERAADA, German Registry of Acute Aortic Dissection Type A score; RENAL, a score for renal mass complexity; PADUA, a score for renal mass complexity; SURPAS, Surgical Risk Preoperative Assessment System; AiTOR, AI model based on Toronto patient outcome data; POTTER, Predictive Optimal Trees in Emergency Surgery Risk.

### Surgeon prediction of mortality

A total of 13 studies reporting the predictive accuracy of surgeon gestalt for mortality were identified. Of these studies, nine directly asked surgeons to predict postoperative mortality and were included in the analysis (see *Table 2*). The remaining four studies were excluded as they correlated estimates of overall adverse outcomes (Woodfield 2007, Burgos 2008), patient survival time (Smith 2008), or fitness for open surgery (George 2020) with mortality. All nine of the included studies reported surgeon prediction of ‘operative’ mortality, the definition of which varied. Four studies defined this as solely including deaths within 30 days (Hobson 2007, Dyas 2022, El Moheb 2023, Gwilym 2022/2024), two included deaths beyond 30 days if directly caused by surgical complications (Cornwell 2012, Jain 2014), and three included all deaths within 30 days or within index hospitalization (Pons 1999, Bakaeen 2010, Wu 2023). Further variation was introduced as two studies asked surgeons to predict from vignettes without seeing the patient (Dyas 2022, El Moheb 2023). Three reports did not include confidence intervals (Pons 1999, Hobson 2007, Gwilym 2022).

**Table 2:** Predictive accuracy of surgeons and scoring tools for mortality

Report	True rate (%)	Gestalt			Scoring tool		
		Timing	Mean calibration (95% c.i.)	C-statistic (95% c.i.)	Tool used	Mean calibration (95% c.i.)	C-statistic (95% c.i.)
**30-day follow-up**							
Pons 1999^SR4^	11	Preop	NR	0.70 (NR)	Novel model	NR	0.76 (NR)
Hobson 2007^SR8^	9	Preop	NR	0.90 (NR)	POSSUM, P-POSSUM	NR	0.95 (NR), 0.94 (NR)
Bakaeen 2010^SR13^	5	Preop	1.54 (1.37,1.71)	0.73 (NR)	CICSP	1.22 (1.06,1.39)	0.75 (NR)
Cornwell 2012^SR14^	6	Preop	1.97 (1.84,2.09)	NR	CICSP	1.23 (1.03,1.43)	NR
Jain 2014^SR15^	3	Preop	1.64 (1.60,1.67)	0.73 (0.69,0.77)	VA CICSP	1.30 (1.27,1.33)	0.78 (0.75,0.82)
Dyas 2022^SR33^	7	Preop	0.68 (0.62,0.74)	0.85 (NR)	SURPAS	1.72 (1.43,2.02)	0.98 (NR)
Wu 2023^SR28^	10	Preop	NR	0.71 (0.59,0.83)	GERAADA	NR	0.74 (0.63,0.85)
El Moheb 2023^SR35^	17	Preop	2.19 (NR)	0.84 (0.76,0.92)	POTTER	0.98 (*nr*)	0.88 (0.83,0.93)
Gwilym 2022^SR29^	10	Preop	1.81 (NR), 2.16 (NR)*	0.79 (NR), 0.70 (NR)*	Multiple†	Multiple†	Multiple†
**180-day follow-up**							
Cornwell 2012^SR14^	11	Preop	1.09 (1.02,1.16)	NR	CICSP	0.68 (0.57,0.79)	NR
**1-year follow-up**							
Jain 2014^SR15^	7	Preop	0.76 (0.74,0.78)	0.61 (NR)	VA CICSP	0.61 (0.59,0.62)	0.72 (NR)
Gwilym 2024^SR30^	28	Preop	1.24 (NR), 1.30 (NR)*	0.73 (0.66,0.79), 0.72 (0.65,0.79)*	Multiple†	Multiple†	Multiple†
**5-year follow-up**							
Jain 2014^SR15^	19	Preop	0.29 (0.29,0.30)	0.64 (NR)	VA CICSP	0.23 (0.23,0.24)	0.72 (NR)

^*^The first value represents consultant predictions and the second value represents trainee predictions. †Too many scoring tools used to be listed in main table; see *Table S5* for details. NR, not reported; POSSUM, Physiological and Operative Severity Score for the Enumeration of Mortality and Morbidity; P-POSSUM, Portsmouth Physiological and Operative Severity Score for the Enumeration of Mortality and Morbidity; CICSP, Continuous Improvement in Cardiac Surgery Program; VA, Veterans Affairs; SURPAS, Surgical Risk Preoperative Assessment System; GERAADA, German Registry of Acute Aortic Dissection Type A score; POTTER, Predictive Optimal Trees in Emergency Surgery Risk.

#### Gestalt accuracy for mortality

**Fig. 2:**
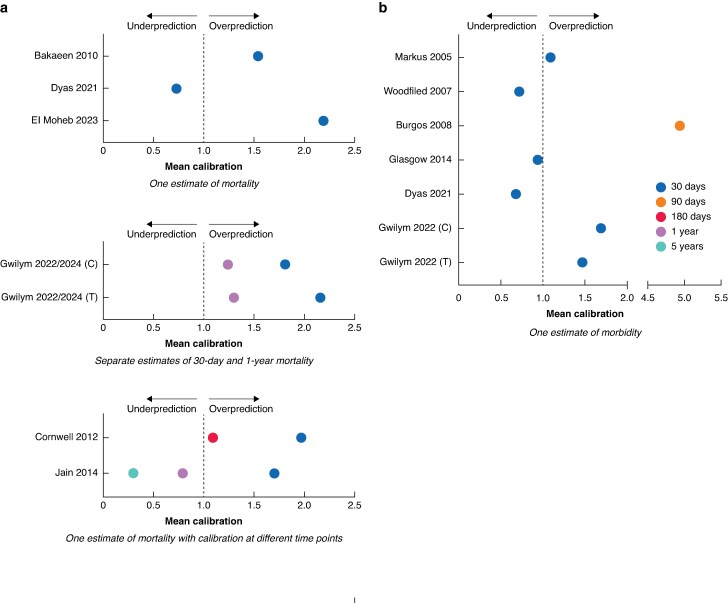
Mean calibration **a** Mean calibration of surgeon estimates of mortality. **b** Mean calibration of surgeon estimates of total morbidity. C, consultant; T, trainee.

A total of eight studies reported surgeon gestalt discrimination for operative mortality, with C-statistics ranging from 0.70 to 0.90. *Table 2* shows that gestalt discrimination was ‘acceptable’ in five studies, ‘excellent’ in two studies, and ‘outstanding’ in one study. *Figure 2a* shows the mean calibration from the six studies in which it could be calculated; in all of the studies, except Dyas 2022, surgeons overestimated operative mortality risk. The studies with the most extreme mean calibration values (0.73 in Dyas and 2.19 in El Moheb) both asked surgeons to predict based on vignettes and one had a small sample size of 30 cases (Dyas 2022).

#### Gestalt accuracy for mortality changes with prediction time frame

A total of three studies reported the accuracy of surgeon gestalt at both 30 days and at least one later time point. Of these studies, two asked surgeons to make a single prediction without specifying a time frame, then assessed accuracy for different time points (Cornwell 2012, Jain 2014). The other study asked surgeons to make two independent predictions, one specific for 30 days and the other for 1 year (Gwilym 2022/2024). In Cornwell 2012 and Gwilym 2022/2024, surgeons overestimated 30-day mortality, but calibration improved at 180 days and 1 year respectively (see *Fig. 2a*). In Jain 2014, surgeons overestimated 30-day mortality, but underestimated mortality at 1 year and 5 years. In the two studies in which it was reported, discrimination was worse at 1 year than it was at 30 days.

#### Can scoring tools better predict operative mortality compared with surgeon gestalt?

All nine studies compared predictions of operative mortality made by surgeons with those made by scoring tools. A variety of tools were used, ranging from some near-universal tools (for example Surgical Outcome Risk Tool SORT) and Surgical Risk Preoperative Assessment System (SURPAS)) to those specific to a specialty or procedure. In seven studies, tools were subjectively selected by the investigators, and, in one study (Pons 1999), a novel tool was developed using data from the same study population. The remaining study, Gwilym 2022/2024, systematically identified all tools developed for predicting mortality following lower limb amputation and therefore included a large number of tools. In all studies, at least one tool exhibited better discrimination for operative mortality than surgeons. Where tools outperformed surgeons, the mean improvement in the C-statistic was 0.05. The greatest improvement compared with surgeon gestalt was 0.13 in a vignette-based study (Dyas 2022). Five studies found that the improved accuracy of scoring tools over gestalt was statistically significant (Pons 1999, Jain 2014, Dyas 2022, Wu 2023, El Moheb 2023).

### Surgeon prediction of morbidity

A total of 24 studies investigated the predictive accuracy of surgeon gestalt for morbidity (see *Table 3*). Of these studies, four used indirect estimates and were excluded (Kaafarani 2005, Ulyett 2015, Kumar 2017, George 2020). Also, four studies considered predictions of a specific complication rather than total morbidity and were analysed separately (see *Table S6*). These latter studies reported moderate discrimination and calibration of both surgeon gestalt and scoring tool assessments.

**Table 3:** Predictive accuracy of surgeons and scoring tools for morbidity

Report	True rate (%)	Gestalt			Scoring tool		
		Timing	Mean calibration (95% c.i.)	C-statistic (95% c.i.)	Tool used	Mean calibration (95% c.i.)	C-statistic (95% c.i.)
**30-day follow-up**							
Markus 2005^SR6^	30	Postop	1.09 (NR)	NR	POSSUM	1.57 (NR)	NR
Woodfield 2007^SR9^	34	Preop, postop	0.72 (0.69,0.75), 0.77 (0.73,0.80)	0.64, 0.65 (NR)	Otago Audit Model	NR	0.76 (NR)
Glasgow 2014^SR16^	8	Preop	0.94 (NR)	NR	Novel model	1.10 (NR)	NR
Woodfield 2017^SR21^	24	Preop, postop	NR	0.78 (0.73,0.82), 0.81 (0.77,0.85)	POSSUM, P-POSSUM	NR	0.76 (0.70,0.80), 0.84 (0.79,0.86)
Samim 2018^SR22^	56	Preop	NR	0.64 (0.58,0.71)	Donati	NR	0.62 (0.56,0.68)
Dyas 2022^SR33^	33	Preop	0.68 (0.62,0.74)	0.76 (NR)	SURPAS	0.84 (0.75,0.92)	0.84 (NR)
Marwaha 2023^SR27^	24	Preop	NR	0.70 (0.62,0.78)	NSQIP	NR	0.83 (0.80,0.85)
Gwilym 2022^SR29^	19	Preop	1.69 (NR), 1.47 (NR)*	0.66 (NR), 0.62 (NR)*	Wied	NR	0.52 (NR)
Berrigan 2025^SR31^	27	Preop	NR	0.71 (0.63,0.78)	Novel model	NR	0.84 (0.78,0.88)
**90-day follow-up**							
Burgos 2008^SR11^	10	NR	4.94 (4.67,5.21)	0.83 (0.76,0.91)	POSSUM, Goldman, Charlson	2.29 (2.20,2.39), NR, NR	0.73 (0.62,0.84), 0.65 (0.52,0.78), 0.71 (0.60,0.81)
Farges 2014^SR17^	49	Preop, postop	NR	0.77 (0.69,0.94), 0.78 (0.69,0.95)	Novel model	NR	0.80 (0.69,0.93)

^*^The first value represents consultant predictions and the second value represents trainee predictions. NR, not reported; POSSUM, Physiological and Operative Severity Score for the Enumeration of Mortality and Morbidity; P-POSSUM, Portsmouth Physiological and Operative Severity Score for the Enumeration of Mortality and Morbidity; SURPAS, Surgical Risk Preoperative Assessment System; NSQIP, National Surgical Quality Improvement Program.

Of the remaining 16 studies, 11 reported either gestalt assessment C-statistic or calibration data so were included in the analysis. Of these 11 studies, 9 considered ‘operative’ morbidity, with 8 studies defining this as solely including complications within 30 days (Markus 2005, Woodfield 2007, Glasgow 2014, Woodfield 2017, Dyas 2022, Marwaha 2023, Berrigan 2025, Gwilym 2022) and 1 study also including later complications provided they occurred during index hospitalization (Samim 2018). The remaining two studies reported morbidity within 90 days (Burgos 2008, Farges 2014). Definitions of morbidity varied, with six studies considering all adverse events including death (Woodfield 2007, Burgos 2008, Glasgow 2014, Woodfield 2017, Marwaha 2023, Berrigan 2025), three studies specifying complications of a particular Clavien–Dindo classification (Samim 2018, Farges 2014, Gwilym 2022/2024), and two studies considering a list of complications predefined by the study authors (Markus 2005, Dyas 2022). Three reports did not include confidence intervals (Markus 2007, Glasgow 2014, Gwilym 2022).

#### Gestalt accuracy for morbidity

A total of seven studies reported surgeon gestalt discrimination for operative morbidity. C-statistics were generally lower than for mortality, ranging from 0.64 to 0.78. *Figure 2b* shows the mean calibration from six studies, of which five predicted operative morbidity and one predicted 90-day morbidity. Of the six studies, two reported a mean calibration close to 1 (Markus 2005, Glasgow 2014), two reported that surgeons were underpredicting morbidity (Woodfield 2007, Dyas 2022), and two showed an overprediction (Burgos 2008, Gwilym 2022). The study that showed the most substantial overestimation (mean calibration 4.94) had a small surgeon sample size of three (Burgos 2008).

#### Can scoring tools better predict operative morbidity compared with surgeon gestalt?

A total of nine studies compared predictions of operative morbidity made by surgeons with predictions made by scoring tools. Of these studies, seven reported discrimination of both gestalt and scoring tools, and mean calibration could be calculated in three. Scoring tools had better discrimination in five studies and worse discrimination in two. The two studies with tools with worse discrimination than surgeons had especially low C-statistics (0.52 in Gwilym 2022 and 0.62 in Samim 2018). Where mean calibration could be calculated, surgeons performed better than tools in two of the studies and worse in the third, which used a vignette-based design (Dyas 2022). No study found a statistically significant difference between gestalt and scoring tool accuracy.

### Combining surgeon gestalt and scoring tool predictions

A total of five studies tested integrated models incorporating predictions made by both surgeon gestalt and scoring tools (Woodfield 2007, Gwilym 2022/2024, Wu 2023, Marwaha 2023, Ishikita 2024) (see *Table 4*). All of these studies reported integrated model C-statistics and one reported mean calibration, but only three reported confidence intervals (Wu 2023, Marwaha 2023, Ishikita 2024). In all studies, the integrated model had better discrimination than the gestalt-blind scoring tool, and, in the one study in which it was reported, better mean calibration (Gwilym 2022). In three studies, the integrated tool showed better discrimination than surgeon gestalt (Wu 2023, Marwaha 2023, Woodfield 2007). In Gwilym 2022, whilst the integrated tool mean calibration was better than surgeon gestalt, SORT-2 underpredicted mortality overall, whereas surgeons tended to overpredict. On average, the C-statistic for integrated tools was 0.07 higher than gestalt and 0.04 higher than a gestalt-blind tool.

**Table 4:** Predictive accuracy of integrated tools

Report	Tool used	Gestalt		Scoring tool		Integrated model	
		C-statistic (95% c.i.)	Mean calibration (95% c.i.)	C-statistic (95% c.i.)	Mean calibration (95% c.i.)	C-statistic (95% c.i.)	Mean calibration (95% c.i.)
**Mortality**							
Wu 2023^SR28^	GERAADA	0.71 (0.59,0.83)	NR	0.74 (0.63,0.85)	NR	0.76 (0.64,0.88)	NR
Gwilym 2022^SR29^	SORT	0.79 (NR), 0.70 (NR)*	1.81 (NR)	0.72 (NR)	0.55 (NR)	0.77 (NR)	0.85 (NR)
**Morbidity**							
Marwaha 2023^SR27^	NSQIP	0.72 (0.64,0.80), 0.56 (0.41,0.72)*	NR	0.83 (0.80,0.85)	NR	0.86 (0.79,0.93), 0.87 (0.77,0.97)*	NR
Woodfield 2007^SR9^	Otago Audit Model	0.64 (NR), 0.65 (NR)†	0.72 (NR), 0.77 (NR)†	0.76 (NR)	NR	0.77 (NR)	NR
**Major adverse cardiovascular event**							
Ishikita 2024^SR34^	AiTOR	0.92 (0.72,0.98)	NR	0.86 (0.58,0.96)	NR	0.92 (0.72,0.98)	NR

^*^The first value represents consultant predictions and the second value represents trainee predictions. †The first value represents preoperative predictions and the second value represents postoperative predictions. GERAADA, German Registry of Acute Aortic Dissection Type A score; NR, not reported; SORT, Surgical Outcome Risk Tool; NSQIP, National Surgical Quality Improvement Program score; AiTOR, AI model based on Toronto patient outcome data.

A further study demonstrated that unblinding surgeons to prediction by the POTTER tool improved the discrimination of surgeon predictions of mortality in vignette-based cases (El Moheb 2023). Both tool-blind and tool-informed gestalt predictions exhibited worse discrimination than the tool alone.

### Surgeon gestalt and experience

A total of six studies compared gestalt predictions made by consultant surgeons and trainees. Four of these reported C-statistics, with one study being described in two separate reports (see *Table 5*). A single report did not include confidence intervals (Gwilym 2022). Gwilym 2022/2024 compared consultant and trainee predictions for a range of adverse postoperative outcomes at 30 days and 1 year, and found that consultants showed better discrimination than trainees in every category. The magnitude of these differences in C-statistics ranged from 0.01 for 1-year mortality to 0.11 for 30-day revision surgery. Marwaha 2023 and Berrigan 2025 also reported that consultant predictions for morbidity had better discrimination compared with trainees.

**Table 5:** Predictive accuracy of consultants *versus* trainees

Report	Prediction	Consultant C-statistic (95% c.i.)	Trainee C-statistic (95% c.i.)	Consultant mean calibration (95% c.i.)	Trainee mean calibration (95% c.i.)
Woodfield 2017^SR21^	30-day morbidity	0.76 (0.71,0.85)	0.82 (0.77,0.87), 0.85 (0.78,0.93)*	NR	NR
Gwilym 2022^SR29^	30-day mortality	0.79 (NR)	0.70 (NR)	1.81 (NR)	2.16 (NR)
	30-day morbidity	0.66 (NR)	0.62 (NR)	1.69 (NR)	1.47 (NR)
	30-day revision	0.81 (NR)	0.70 (NR)	2.16 (NR)	1.95 (NR)
Gwilym 2024^SR30^	1-year mortality	0.73 (0.66,0.79)	0.72 (0.65,0.79)	1.24 (NR)	1.30 (NR)
	1-year revision	0.63 (0.54,0.73)	0.61 (0.52,0.71)	1.48 (NR)	1.65 (NR)
Marwaha 2023^SR27^	30-day morbidity	0.72 (0.64,0.80)	0.56 (0.41,0.72)	NR	NR
Berrigan 2025^SR31^	30-day morbidity	0.71 (0.61,0.78)	0.62 (0.41,0.80)	NR	NR

^*^The first value represents senior registrar predictions and the second value represents junior registrar predictions. NR, not reported.

Conversely, Woodfield 2017 found that predictive accuracy and surgeon experience were negatively correlated, with junior registrars outperforming senior registrars, and both outperforming consultants. This study also reported that gestalt prediction may improve with dedicated study. Surgeons were asked to predict general morbidity for a set of patients. The surgeons were then told the true outcomes and given feedback before being asked to predict general morbidity on a second set of patients. After feedback, the C-statistic increased from 0.78 (95% c.i. 0.73 to 0.82) to 0.90 (95% c.i. 0.83 to 0.97).

### Surgeon gestalt and operation type

A total of three studies stratified surgeon predictions by operation urgency. Of these studies, one found that gestalt assessment overestimated mortality for elective procedures, but underestimated mortality for emergency procedures (mean calibration 1.29 *versus* 0.79); additionally, gestalt calibration was better than Physiological and Operative Severity Score for the Enumeration of Mortality and Morbidity (POSSUM) for elective procedures, but worse for emergency procedures (Markus 2005). Another study found that surgeons overestimated mortality for both elective and urgent procedures, but overestimated to a greater extent for elective procedures (Cornwell 2012). The final study found that gestalt assessment had better discrimination for morbidity in elective procedures compared with urgent procedures (C-statistic 0.71 *versus* 0.65) (Marwaha 2023).

A single study classified surgeon predictions by surgical specialty (Woodfield 2017). The study authors observed that predictions made by colorectal, upper gastrointestinal, and vascular surgeons had C-statistics of 0.75, 0.83, and 0.84 respectively when predicting 30-day morbidity. Patients were not evenly split between specialties; there were 718 colorectal patients, 200 upper gastrointestinal patients, and 163 vascular patients.

## Discussion

This review investigated the predictive accuracy of surgeon gestalt assessment, synthesizing data from 33 657 patients across 34 studies. This is the most comprehensive review on surgeon gestalt assessment to date and identifies ten studies not included in the previous most recent review^22^. Overall, surgeons were good at predicting postoperative mortality, as demonstrated by high C-statistics, ranging from acceptable to outstanding. This aligns with similar observations in other specialties of medicine—in general practice, clinician ‘gut feelings’ have been reported to predict disease outcomes even without definitive diagnostic or prognostic criteria^30,31^.

Surgeons consistently overestimated operative mortality risk. This may be due to surgeons instinctively accounting for longer-term outcomes when asked to predict 30-day mortality, resulting in poor calibration within the specified time frame, but better calibration at later time points. This pattern was observed in three of the included studies^SR14,SR15,SR29,SR30^. Alternatively, overestimating risk may be done for the purpose of safety netting, with excess caution due to the potentially high stakes of any operation. Pessimistic predictions may also reflect the disproportionate emotional impact of a postoperative death—surgeons may more readily remember a patient who died than patients who had an uneventful clinical course.

Surgeon gestalt predictions of morbidity did not follow the same pattern of overestimation as mortality. Although one study reported an extreme mean calibration (4.94), the study authors considered 90-day outcomes rather than operative morbidity and collected data from just three surgeons. Gestalt prediction of morbidity also had lower discrimination compared with mortality. This pattern was seen in studies that assessed the predictive accuracy for both mortality and morbidity, so is less likely to be due to differences in study design^SR29,SR33^. Morbidity risk may be more difficult to estimate compared with mortality as it requires surgeons to consider a much wider range of possible outcomes. This is supported by one study that found surgeons were much more accurate when directed to predict a specific complication instead of overall morbidity^SR9^.

Risk scoring tools showed good accuracy with several tools outperforming surgeons in both discrimination and calibration. Whilst some studies found statistically significant improvements for risk scoring tools compared with gestalt, effect sizes tended to be small and it is unclear if the differences are clinically significant. General purpose scoring tools such as SORT and SURPAS performed particularly well, as did some specialty-specific tools such as POSSUM and Continuous Improvement in Surgery Program score (CICSP). However, the overall mean difference between gestalt and scoring tools was small due to a number of particularly poorly predictive tools listed in Samim 2018 and Gwilym 2022/2024. The AI tools were unremarkable. Although both POTTER and AI model based on Toronto patient outcome data (AiTOR) had good discrimination, they performed modestly in the context of other scoring tools^SR34,SR35^. Additionally, whilst POTTER outperformed surgeon gestalt, AiTOR performed worse. Analysis was limited as only two of the included studies tested AI tools; however, these data align with current literature, with one systematic review finding that AI tools are currently non-superior to scoring tools developed with logistic regression^32^.

The marginally better accuracy of scoring tools observed in these studies may be due to several factors. First, it is possible these findings represent a true superiority of modern scoring tools over gestalt for surgical risk assessment. However, many tools are designed specifically to predict outcomes within a 30-day time frame, compared with surgeons who may consider longer-term outcomes. Some studies also tested novel tools developed and validated in the same population, giving them an unfair advantage. This was shown in one study, where initial tests showed the scoring tool significantly outperforming surgeons; however, when the tool was retested in a separate validation cohort, statistical significance was lost^SR4^. Additionally, some studies asked surgeons to predict patient outcomes from clinical case vignettes, limiting the number of variables they could consider. Surgeons may also consider variables not included in scoring tools. Two studies investigated surgeon notes and found that discrepancies between gestalt and scoring tool assessment were most often due to the selection of different variables^SR13,SR14^.

The flexibility of gestalt has both potential benefits and pitfalls. Without the constraints of a scoring tool, good clinicians may consider factors that are not generally predictive of risk on a population level and would thus be missed by a logistic regression model, but may be pertinent for specific patients. On the other hand, some clinicians may be better at selecting appropriate variables than others, so the quality of gestalt assessment may be inconsistent. Nevertheless, maintaining good gestalt is clinically valuable. A recent multicentre study showed that utilizing AI assistance in colonoscopy led to overall poorer standards in adenoma detection, most likely due to clinician deskilling^33^. Thus, surgeons should be cautious about relying too heavily on risk scoring tools to replace their own predictions.

This review found that integrating gestalt assessment into scoring tools can improve accuracy. These composite models incorporate both the flexibility of surgeon gestalt to select and weigh variables differently from patient to patient and the standardized algorithms of a systematic scoring tool. There is some precedence for this. For example, clinician ‘gut feeling’ is a highly weighted variable in the Wells’ score^34^. Clinician gestalt is also required for the SORT-2 score, which significantly outperforms the gestalt-blind SORT score^19^. However, although several studies showed integrated models outperforming surgeon gestalt and scoring tools, effect sizes were generally small and two studies did not report confidence intervals. It is also unclear if the observed superiority was a fair representation in all studies. One study incorporated surgeon predictions into the Otago Audit Model, but then further optimized this new composite tool in the same patient population used for validation^SR9^. This would have given the integrated model an unfair advantage over non-optimized prediction modalities. Overall, more data are needed to investigate whether the improved accuracy of integrated tools is clinically meaningful.

Clinicians with more experience would reasonably be expected to be better at pattern recognition and predicting outcomes. However, this association has been observed to be less clear for other healthcare professionals; studies show that senior nurses rely more on gestalt than juniors, despite no evidence that they make better predictions^35–38^. One potential explanation for this is that clinical gestalt is determined more by disposition, personality type, and life experience than medical knowledge or clinical skill. Nevertheless, in this review, of the four studies stratifying predictions by surgeon experience, three found that risk estimates made by consultants had better discrimination than those made by trainees, albeit with varying effect size^SR27,SR29,SR30,SR31^. Interestingly in Gwilym 2022/2024, although the increased accuracy of consultants over trainees was small when predicting mortality and morbidity, there was a much bigger difference when predicting need for revision amputation surgery. This may be because judging whether a patient may need another operation requires very specialist knowledge and is thus more familiar to surgeons with greater clinical experience. The remaining study found trainee predictions outperformed consultant predictions^SR21^. However, this study was limited by a small sample size, with 92% of predictions made by just 24 surgeons. Interestingly, the study authors also observed that dedicated study may affect gestalt accuracy, which warrants further exploration. The study reported a sizeable improvement in discrimination from ‘acceptable’ to ‘outstanding’ after feedback.

Gestalt assessment accuracy may differ between procedure types. Two studies found that surgeons were better at predicting complications for elective procedures than urgent procedures^SR6,SR27^. One contributing factor could be the relatively controlled environment of elective surgeries, facilitating systematic and logical weighing up of variables; conversely, emergency surgeries may be more time-pressured. Surgeons may also have access to more information for elective surgeries, collated from outpatient clinical encounters and referral letters, whereas they may often be meeting patients for the first time in the case of emergency surgeries. There is also some evidence that surgeons overestimate risk more for elective procedures than for urgent procedures^SR6,SR14^. One contributing factor could be surgeons placing a greater emphasis on safety netting in elective scenarios. Surgeons may unconsciously exaggerate risk for planned surgeries, reflecting greater hesitancy to operate on a patient when the situation is not urgent. Additionally, lack of proper patient workups in emergency situations may lead to underestimating risk. One study also found that gestalt discrimination varied between specialties^SR21^. This may be due to a variety of reasons, such as differences in training, or some specialties dealing with more unpredictable procedures than others. More data are needed to elucidate whether the predictive accuracy of gestalt is consistently different between specialties and why this might be.

Drawing definitive conclusions from these data is challenging due to significant heterogeneity. There was substantial variation in the specific question that surgeons were asked. Whilst many studies asked for direct estimates of complication risk, others opted for more indirect questions about patient frailty, operation difficulty, or surgeon frustration^SR7,SR24,SR32^. There was also often a lack of clarity about whether predictions were being made for a particular time frame. For example, for studies investigating operative morbidity, some asked surgeons specifically about 30-day risk^SR29^, whereas others asked for a generalized estimate then followed patients up for 30 days^SR14,SR15^. Definitions of ‘mortality’ and ‘morbidity’ were inconsistent, with differences in what constituted an ‘operative’ complication and whether ‘morbidity’ included all adverse events or only those of a certain severity. Additionally, there was variation in how surgeon prediction was quantified. Methods included percentage or graded estimates, binary predictions about whether or not a complication would happen, and use of a Visual Analogue Scale. A large range of reference tests was used, each with varying degrees of predictive value. Some were well-established scoring tools frequently used in clinical practice such as POSSUM; others were less appraised or even entirely novel at the time of the study. Furthermore, there was heterogeneity in statistical analysis, especially in studies drawing comparisons between surgeon gestalt and scoring tool predictions. Some studies also did not report confidence intervals, limiting the conclusions that may be drawn from their data^SR13,SR29,SR33^. Additionally, although some studies found statistically significant differences, it was often unclear if these differences were also clinically meaningful. Finally, exploring how different surgeon characteristics might affect gestalt was difficult. Whilst all studies reported patient sample size, few reported the number of surgeons involved. Detailed breakdowns of surgery features were also rarely given, limiting analysis on how gestalt may vary depending on different factors such as surgical specialty or operation urgency.

Future studies should draw lessons from these limitations to improve quality of analysis and reduce risk of bias. In terms of next steps, factors contributing to effective gestalt have been minimally explored and may be an interesting avenue for future research. This could be done by stratifying surgeons by various characteristics, such as personality type. Additionally, studies should endeavour to investigate not only whether the difference between gestalt and scoring tool predictive accuracy is statistically significant but also clinically meaningful. This could be done by defining clear minimal clinically important differences (MCIDs) or by measuring the number needed to predict (NNP) or the absolute risk difference (ARD) between gestalt and scoring tool assessment groups. Important items for future studies to report include surgeon sample size, specific time frames, and gestalt assessment C-statistic and calibration. Studies should also ensure to use separate training and validation populations when developing new prediction models. Furthermore, clarifying the exact question posed to surgeons and asking surgeons to record which variables they considered to reach a prediction would allow greater transparency and facilitate good analysis.

This review suggests surgeon gestalt can play an important role in predicting surgical outcomes. Furthermore, the value of gestalt should not be overlooked in the development of new risk scoring tools. Hospitals may consider introducing databases to capture and compile surgeon predictions of adverse postoperative events, similar to the Veterans Affairs Continuous Improvement in Cardiac Surgery Program (VA CICSP) system. This information would be useful for the development of new prediction models, as well as for quality improvement schemes. To maximize the quality of their risk assessments, surgeons should consider both their own gestalt predictions and scoring tool outputs. They should also consult other clinicians who may value different variables and bring fresh perspectives, particularly more experienced colleagues. Finally, surgeons should maintain reflective practice, deliberate the outcomes of previous cases, and continuously seek ways to improve the accuracy of their predictions.

## Supplementary Material

znaf249_Supplementary_Data

## Data Availability

Data are available on the Open Science Framework at DOI: 10.17605/OSF.IO/TZNU5.
