# Supplementary material for: Predictive accuracy of surgeon gestalt for adverse postoperative outcomes: systematic review
Source: Br J Surg. 2025 Nov 29;112(12):znaf249. doi: 10.1093/bjs/znaf249 (PMC12663761; doi:10.1093/bjs/znaf249)
Supplement: znaf249_Supplementary_Data [file znaf249_supplementary_data.docx]

**Predictive accuracy of surgeon gestalt for adverse postoperative outcomes – a systematic review**

Jun J. Yang^a^, Samuel J. Mullan^a^, Tahmid M. Rayhan^a^, Ciaran K. S. S. Sandhu^a^, Anshu N. Ramaiya^a^, Carl J. Heneghan^b,c^

^a^School of Medicine and Biomedical Sciences, University of Oxford, John Radcliffe Hospital

^b^Nuffield Department of Primary Healthcare Sciences, University of Oxford

^c^Centre for Evidence Based Medicine, University of Oxford

Correspondence to: Jun J Yang (email: [jonnyyang51@gmail.com](mailto:jonnyyang51@gmail.com)) (ORCID:

**Supplementary Materials - Index**

| **Supplementary Methods** |  |
| --- | --- |
| Supp Table 1: PRISMA Checklist | *pag. 2* |
| Supp Table 2: Search Strategy  Supp Table 3: Extraction Items | *pag. 5*  *pag. 7* |
| **Supplementary Results** |  |
| Supp Table 4: QUADAS-2 Risk of Bias | *pag. 8* |
| Supp Table 5: Additional Scoring Tools  Supp Table 6: Specific Complications | *pag. 9 pag. 10* |
| **References** |  |
| References for ‘Supp Table 5’ | *pag. 11* |
| Supp Table 7: Supplementary References | *pag. 13* |

**Supplementary Methods**

Supplementary Table 1: PRISMA checklist

| **Section and Topic** | **Item #** | **Checklist item** | **Location where item is reported** |
| --- | --- | --- | --- |
| **TITLE** | | |  |
| Title | 1 | Identify the report as a systematic review. | Page 1 |
| **ABSTRACT** | | |  |
| Abstract | 2 | See the PRISMA 2020 for Abstracts checklist. | Page 2 |
| **INTRODUCTION** | | |  |
| Rationale | 3 | Describe the rationale for the review in the context of existing knowledge. | Page 3 |
| Objectives | 4 | Provide an explicit statement of the objective(s) or question(s) the review addresses. | Page 3 |
| **METHODS** | | |  |
| Eligibility criteria | 5 | Specify the inclusion and exclusion criteria for the review and how studies were grouped for the syntheses. | Page 4 |
| Information sources | 6 | Specify all databases, registers, websites, organisations, reference lists and other sources searched or consulted to identify studies. Specify the date when each source was last searched or consulted. | Page 4 |
| Search strategy | 7 | Present the full search strategies for all databases, registers and websites, including any filters and limits used. | Supplementary Table 2 |
| Selection process | 8 | Specify the methods used to decide whether a study met the inclusion criteria of the review, including how many reviewers screened each record and each report retrieved, whether they worked independently, and if applicable, details of automation tools used in the process. | Page 4 |
| Data collection process | 9 | Specify the methods used to collect data from reports, including how many reviewers collected data from each report, whether they worked independently, any processes for obtaining or confirming data from study investigators, and if applicable, details of automation tools used in the process. | Page 5 |
| Data items | 10a | List and define all outcomes for which data were sought. Specify whether all results that were compatible with each outcome domain in each study were sought (e.g. for all measures, time points, analyses), and if not, the methods used to decide which results to collect. | Supplementary Table 3 |
|  | 10b | List and define all other variables for which data were sought (e.g. participant and intervention characteristics, funding sources). Describe any assumptions made about any missing or unclear information. | Supplementary Table 3 |
| Study risk of bias assessment | 11 | Specify the methods used to assess risk of bias in the included studies, including details of the tool(s) used, how many reviewers assessed each study and whether they worked independently, and if applicable, details of automation tools used in the process. | Page 5 |
| Effect measures | 12 | Specify for each outcome the effect measure(s) (e.g. risk ratio, mean difference) used in the synthesis or presentation of results. | Page 5 |
| Synthesis methods | 13a | Describe the processes used to decide which studies were eligible for each synthesis (e.g. tabulating the study intervention characteristics and comparing against the planned groups for each synthesis (item #5)). | Page 5 |
|  | 13b | Describe any methods required to prepare the data for presentation or synthesis, such as handling of missing summary statistics, or data conversions. | Page 5 |
|  | 13c | Describe any methods used to tabulate or visually display results of individual studies and syntheses. | Page 5 |
|  | 13d | Describe any methods used to synthesize results and provide a rationale for the choice(s). If meta-analysis was performed, describe the model(s), method(s) to identify the presence and extent of statistical heterogeneity, and software package(s) used. | Page 4 |
|  | 13e | Describe any methods used to explore possible causes of heterogeneity among study results (e.g. subgroup analysis, meta-regression). | Page 5 |
|  | 13f | Describe any sensitivity analyses conducted to assess robustness of the synthesized results. | n/a |
| Reporting bias assessment | 14 | Describe any methods used to assess risk of bias due to missing results in a synthesis (arising from reporting biases). | n/a |
| Certainty assessment | 15 | Describe any methods used to assess certainty (or confidence) in the body of evidence for an outcome. | Page 5 |
| **RESULTS** | | |  |
| Study selection | 16a | Describe the results of the search and selection process, from the number of records identified in the search to the number of studies included in the review, ideally using a flow diagram. | Figure 1 |
|  | 16b | Cite studies that might appear to meet the inclusion criteria, but which were excluded, and explain why they were excluded. | n/a |
| Study characteristics | 17 | Cite each included study and present its characteristics. | Table 1 |
| Risk of bias in studies | 18 | Present assessments of risk of bias for each included study. | Supplementary Table 4 |
| Results of individual studies | 19 | For all outcomes, present, for each study: (a) summary statistics for each group (where appropriate) and (b) an effect estimate and its precision (e.g. confidence/credible interval), ideally using structured tables or plots. | Table 2, 3, 4, 5 |
| Results of syntheses | 20a | For each synthesis, briefly summarise the characteristics and risk of bias among contributing studies. | n/a |
|  | 20b | Present results of all statistical syntheses conducted. If meta-analysis was done, present for each the summary estimate and its precision (e.g. confidence/credible interval) and measures of statistical heterogeneity. If comparing groups, describe the direction of the effect. | Figure 2 |
|  | 20c | Present results of all investigations of possible causes of heterogeneity among study results. | Page 8 |
|  | 20d | Present results of all sensitivity analyses conducted to assess the robustness of the synthesized results. | n/a |
| Reporting biases | 21 | Present assessments of risk of bias due to missing results (arising from reporting biases) for each synthesis assessed. | n/a |
| Certainty of evidence | 22 | Present assessments of certainty (or confidence) in the body of evidence for each outcome assessed. | Page 8 |
| **DISCUSSION** | | |  |
| Discussion | 23a | Provide a general interpretation of the results in the context of other evidence. | Page 10, 11 |
|  | 23b | Discuss any limitations of the evidence included in the review. | Page 12 |
|  | 23c | Discuss any limitations of the review processes used. | Page 12 |
|  | 23d | Discuss implications of the results for practice, policy, and future research. | Page 12 |
| **OTHER INFORMATION** | | |  |
| Registration and protocol | 24a | Provide registration information for the review, including register name and registration number, or state that the review was not registered. | Page 4 |
|  | 24b | Indicate where the review protocol can be accessed, or state that a protocol was not prepared. | Page 4 |
|  | 24c | Describe and explain any amendments to information provided at registration or in the protocol. | Page 4 |
| Support | 25 | Describe sources of financial or non-financial support for the review, and the role of the funders or sponsors in the review. | Page 1 |
| Competing interests | 26 | Declare any competing interests of review authors. | Page 1 |
| Availability of data, code and other materials | 27 | Report which of the following are publicly available and where they can be found: template data collection forms; data extracted from included studies; data used for all analyses; analytic code; any other materials used in the review. | Page 1 |

*From:*  Page MJ, McKenzie JE, Bossuyt PM, Boutron I, Hoffmann TC, Mulrow CD, et al. The PRISMA 2020 statement: an updated guideline for reporting systematic reviews. BMJ 2021;372:n71. doi: 10.1136/bmj.n71. This work is licensed under CC BY 4.0. To view a copy of this license, visit <https://creativecommons.org/licenses/by/4.0/>

Supplementary Table 2: Search Strategy

| Search Strategy | |
| --- | --- |
| MEDLINE code | |
| 1 | “gut-feeling*”[Title/Abstract] OR “gestalt”[Title/Abstract] OR “instinct*”[Title/Abstract] OR “intuition”[Title/Abstract] OR “intuitive”[Title/Abstract] OR “clinical judgement*”[Title/Abstract] OR “surgeon perception*”[Title/Abstract] OR “heuristic*”[Title/Abstract] OR “presentiment*”[Title/Abstract] OR “hunch*”[Title/Abstract] OR “acumen*”[Title/Abstract] OR “surgeon predict*” [Title/Abstract] |
| 2 | “surgeon*”[Title/Abstract] OR “surgery”[Title/Abstract] OR “surgeries”[Title/Abstract] OR “surgical”[Title/Abstract] |
| 3 | “robot*”[Title/Abstract] |
| 4 | 1 AND 2 NOT 3 |
| 5 | 4 AND (systematicreview[Filter]) |
| 6 | 4 AND (casereports[Filter]) |
| 7 | 4 AND (editorial[Filter]) |
| 8 | 4 NOT 5 NOT 6 NOT 7 |
| 9 | 8 AND (English[Filter]) |
| EMBASE code | |
| 1 | (("gut feeling*".tw. OR gestalt.tw. OR instinct*.tw. OR intuition.tw. OR intuitive.tw. OR "clinical judgement*".tw. OR "surgeon perception*".tw. OR heuristic*.tw. OR presentiment*.tw. OR hunch*.tw. OR acumen*.tw.) AND (surgeon*.tw. OR surgery.tw. OR surgeries.tw. OR surgical.tw.)) NOT robot*.tw. |
| 2 | Limit 1 to (conference abstract or conference paper or "conference review" or editorial) |
| 3 | 1 NOT 2 |
| 4 | Limit 3 to english language |
| SCOPUS code | |
| 1 | ((TITLE-ABS("gut feeling*") OR TITLE-ABS(gestalt) OR TITLE-ABS(instinct*) OR TITLE-ABS(intuition) OR TITLE-ABS(intuitive) OR TITLE-ABS("clinical judgement*") OR TITLE-ABS("surgeon perception*") OR TITLE-ABS(heuristic*) OR TITLE-ABS(presentiment*) OR TITLE-ABS(hunch*) OR TITLE-ABS(acumen*)) AND (TITLE-ABS(surgeon*) OR TITLE-ABS(surgery) OR TITLE-ABS(surgeries) OR TITLE-ABS(surgical))) NOT TITLE-ABS(robot*) |
| 2 | 1 exclude [conference paper] AND [editorial] |
| 3 | 2 limit to [English] |
| Clinicaltrials.gov code | |
| 1 | Condition/disease: surgeon OR surgeons Other terms: “gut feeling” OR gestalt OR intuition OR intuitive OR instinct OR clinical judgement OR “surgeon perception” OR heuristic OR presentiment OR hunch OR acumen |
| 2 | Condition/disease: surgeon OR surgeons Intervention/treatment: “gut feeling” OR gestalt OR intuition OR intuitive OR instinct OR clinical judgement OR “surgeon perception” OR heuristic OR presentiment OR hunch OR acumen |
| IEEE Xplore | |
| 1 | ("All Metadata":"gut-feeling*" OR "All Metadata":"gestalt" OR "All Metadata":"instinct*" OR "All Metadata":"intuition" OR "All Metadata":"intuitive" OR "All Metadata":"clinical judgement*" OR "All Metadata":"surgeon perception*" OR "All Metadata":"heuristic*" OR "All Metadata":"presentiment*" OR "All Metadata":"hunch*" OR "All Metadata":"acumen*") AND ("All Metadata":“surgeon*” OR "All Metadata":“surgery” OR "All Metadata":“surgeries” OR "All Metadata":“surgical”) NOT ("All Metadata":"robot") |
| 2 | Apply [journals] AND [magazines] AND [early access articles] |
| ACM digital library | |
| 1 | (("gut-feeling*" OR "gestalt" OR "instinct*" OR "intuition" OR "intuitive" OR "clinical judgement*" OR "surgeon perception*" OR "heuristic*" OR "presentiment*" OR "hunch*" OR "acumen*") AND (“surgeon*” OR “surgery” OR “surgeries” OR “surgical”)) NOT ("robot") |

Supplementary Table 3: Extraction Items

| 1 | First author, year of publication, full title, journal, study design, country(ies), recruitment period, follow-up, number of centres |
| --- | --- |
| 2 | Number of patients in analysis, number of surgeries, number of gestalt predictions, number of scoring tool predictions, age (mean, median, range), male:female ratio, ethnicity(ies), ASA scores, postoperative length of stay |
| 3 | Specific procedure, number of acute/elective/open/laparoscopic/other procedures, surgical specialty(ies) |
| 4 | Number of surgeons, surgeon experience, method of gestalt prediction, assessment timing, assessment type, scoring tool(s) used |
| 5 | Mean surgeon prediction of mortality, mean scoring tool prediction of mortality, surgeon prediction ROC AUC/OR, scoring tool prediction ROC AUC/OR, any significant difference, true mortality rate |
| 6 | Definition of ‘complication’, list of complications reported, mean surgeon prediction of morbidity, mean scoring tool prediction of morbidity, surgeon prediction ROC AUC/OR, scoring tool prediction ROC AUC/OR, any significant difference, true morbidity rate |
| 7 | Mean surgeon prediction of specific complications, mean scoring tool prediction of specific complications, surgeon prediction ROC AUC/OR, scoring tool prediction ROC AUC/OR, any significant difference, true specific complications rate |

**Supplementary Results**

Supplementary Table 4: QUADAS-2

|  | **Risk of bias** | | | | **Applicability Concerns** | | |
| --- | --- | --- | --- | --- | --- | --- | --- |
| **Study** | **Selection** | **Index test** | **Reference** | **Patient flow** | **Selection** | **Index test** | **Reference** |
| **In-person risk assessment** | | | | | | | |
| Pettigrew, 1986 ^SR1^ | + | ++ | ++ | + | - | - | ++ |
| Pettigrew, 1987 ^SR2^ | + | + | + | - | - | - | - |
| Arvidsson, 1996 ^SR3^ | - | + | - | - | - | - | - |
| Pons, 1999 ^SR4^ | - | - | ++ | ++ | - | - | ++ |
| Avidan, 2004 ^SR5^ | ++ | + | ++ | ++ | ++ | ++ | ++ |
| Markus, 2005 ^SR6^ | + | - | - | - | - | + | + |
| Kaafarani, 2005 ^SR7^ | - | - | - | + | - | ++ | - |
| Hobson, 2007 ^SR8^ | + | ++ | - | + | + | - | - |
| Woodfield, 2007 ^SR9^ | ++ | ++ | ++ | + | ++ | - | + |
| Smith, 2008 ^SR10^ | ++ | - | - | + | - | - | - |
| Burgos, 2008 ^SR11^ | - | - | ++ | - | - | - | - |
| Karliczek, 2009 ^SR12^ | - | - | - | - | - | - | - |
| Bakaeen, 2010 ^SR13^ | + | + | ++ | - | - | + | - |
| Cornwell, 2012 ^SR14^ | ++ | ++ | + | ++ | - | ++ | + |
| Jain, 2014 ^SR15^ | - | - | - | - | - | - | - |
| Glasgow, 2014 ^SR16^ | ++ | ++ | ++ | - | - | ++ | + |
| Farges, 2014 ^SR17^ | - | ++ | ++ | - | - | - | - |
| Promberger, 2014 ^SR18^ | - | - | + | + | - | - | + |
| Ulyett, 2015 ^SR19^ | ++ | ++ | + | ++ | - | ++ | + |
| Sammour, 2017 ^SR20^ | - | - | - | + | - | - | - |
| Woodfield, 2017 ^SR21^ | ++ | ++ | ++ | ++ | ++ | ++ | ++ |
| Samim, 2018 ^SR22^ | ++ | - | ++ | + | - | - | - |
| Kohler, 2018 ^SR23^ | + | ++ | + | + | - | - | + |
| George, 2020 ^SR24^ | - | + | - | - | - | ++ | - |
| Vanbrugghe, 2020 ^SR25^ | ++ | - | + | ++ | - | + | + |
| Zaruta, 2023 ^SR26^ | ++ | - | - | ++ | + | - | - |
| Marwaha, 2023 ^SR27^ | ++ | - | - | - | - | - | - |
| Wu, 2023 ^SR28^ | - | + | + | - | - | - | - |
| Gwilym, 2022/2024 ^SR29, SR30^ | - | - | - | - | - | - | - |
| Berrigan, 2025 ^SR31^ | + | - | - | - | + | - | - |
| **Vignette risk assessment** | | | | | | | |
| Kumar, 2017 ^SR32^ | ++ | - | - | + | + | - | + |
| Dyas, 2021 ^SR33^ | - | - | - | - | - | - | - |
| Ishikita, 2023 ^SR34^ | ++ | - | - | + | ++ | - | - |
| El Moheb, 2023 ^SR35^ | ++ | - | - | - | + | - | - |

Supplementary Table 5: Additional Scoring Tools

| **Outcome predicted** | **Scoring Tool** | **C-statistic** | **Mean calibration** |
| --- | --- | --- | --- |
| **Tools identified in Gwilym 2022/2024** ^SR29, SR30^ |  |  |  |
| **30-day mortality** | Nelson AKA^1^ | 0.79 | 3.97 |
|  | Wong (SORT version 2)^2^ | 0.77 | 0.85 |
|  | Nelson BKA^1^ | 0.77 | 11.51 |
|  | Easterlin^3^ | 0.73 | 8.01 |
|  | Jolissaint^4^ | 0.72 | 6.56 |
|  | Protopapa (SORT version 1)^5^ | 0.72 | 0.55 |
|  | Kim^6^ | 0.70 | 0.59 |
|  | Feinglass, AKA^7^ | 0.67 | 7.02 |
|  | Ambler^8^ | 0.67 | 0.45 |
|  | Tang^9^ | 0.65 | 1.76 |
|  | Feinglass, BKA^7^ | 0.62 | 4.72 |
|  | Franchin^10^ | 0.64 | 1.18 |
|  | Patterson^11^ | 0.55 | 1.63 |
| **30-day morbidity** | Wied^12^ | 0.52 | *nr* |
| **30-day MLLA revision** | Ambler^8^ | 0.68 | 0.81 |
| **1-year mortality** | Norvell^13^ | 0.76 | 0.83 |
|  | Kim^6^ | 0.72 | 0.85 |
|  | Campbell^14^ | 0.65 | 0.21 |
| **1-year MLLA revision** | Czerniecki^15^ | 0.55 | 2.25 |
| **Tools identified in Samim 2015** ^SR22^ |  |  |  |
| **All morbidity** | Donati^16^ | 0.62 | *nr* |
| **Morbidity from liver surgery** | Donati^16^ | 0.68 | *nr* |
|  | Breitenstein^17^ | 0.73 | *nr* |
|  | Simons^18^ | 0.57 | *nr* |
|  | Andres^19^ | 0.57 | *nr* |
| **Morbidity from pancreas surgery** | Hill^20^ | 0.55 | *nr* |
|  | Venkat^21^ | 0.55 | *nr* |
|  | Greenblatt^22^ | 0.57 | *nr* |
|  | Ragulin-Coyne^23^ | 0.53 | *nr* |
|  | Uzunoglu^24^ | 0.51 | *nr* |

Supplementary Table 6: Specific Complications

|  |  | **Surgeon gestalt** |  |  | **Scoring tool** |  |  |
| --- | --- | --- | --- | --- | --- | --- | --- |
| **Study** | **True rate %** | **Timing** | **Mean calibration** | **C-statistic** | **Tool used** | **Mean calibration** | **C-statistic** |
| **Anastomotic leakage** |  |  |  |  |  |  |  |
| Karliczek, 2009 ^SR12^ | 14 | Postop | 0.70 | *nr* | Novel model | *nr* | *nr* |
| Sammour, 2016 ^SR20^ | 10 | Postop | *nr* | 0.40 | Novel model | *nr* | 0.84 |
| **Major adverse cardiovascular event** |  |  |  |  |  |  |  |
| Ishikita, 2023 ^SR34^ | 36 | Postop | *nr* | 0.92 | AiTOR | *nr* | 0.86 |
| **Sepsis** |  |  |  |  |  |  |  |
| El Moheb, 2023 ^SR35^ | 8 | Preop | 4.71 | 0.82 | POTTER | 0.73 | 0.82 |
| **Ventilation** |  |  |  |  |  |  |  |
| El Moheb, 2023 ^SR35^ | 14 | Preop | 3.31 | 0.83 | POTTER | 0.92 | 0.92 |
| **Pneumonia** |  |  |  |  |  |  |  |
| El Moheb, 2023 ^SR35^ | 17 | Preop | 2.46 | 0.75 | POTTER | 0.46 | 0.84 |
| **Haemorrhage** |  |  |  |  |  |  |  |
| El Moheb, 2023 ^SR35^ | 40 | Preop | 0.91 | 0.74 | POTTER | 0.41 | 0.84 |
| **Revision surgery** |  |  |  |  |  |  |  |
| Gwilym (30-day) ^SR29^ | 9 | Preop | 2.16, 1.95 * | 0.81, 0.70 * | Ambler | 0.81 | 0.68 |
| Gwilym (1-year) ^SR30^ | 11 | Preop | 1.48, 1.65 * | 0.63, 0.61 * | Czerniecki | 2.25 | 0.55 |

Key –

* First value represents consultant predictions, second value represents trainee predictions

**References**

References for Supplementary Table 5: Additional Scoring Tools

1. Nelson MT, Greenblatt DY, Soma G, Rajimanickam V, Greenberg CC, Kent KC. Preoperative factors predict mortality after major lower-extremity amputation. Surgery. 2012;152(4):685-694; discussion 694-686.

2. Wong DJN, Harris S, Sahni A, Bedford JR, Cortes L, Shawyer R, et al. Developing and validating subjective and objective risk-assessment measures for predicting mortality after major surgery: An international prospective cohort study. PLoS Med. 2020;17(10):e1003253.

3. Easterlin MC, Chang DC, Wilson SE. A practical index to predict 30-day mortality after major amputation. Ann Vasc Surg. 2013;27(7):909-917.

4. Jolissaint JS, Shah SK, Martin MC, Raffetto JD, McPhee JT. Risk prediction of 30-day mortality after lower extremity major amputation. J Vasc Surg. 2019;70(6):1868-1876.

5. Protopapa KL, Simpson JC, Smith NC, Moonesinghe SR. Development and validation of the Surgical Outcome Risk Tool (SORT). Br J Surg. 2014;101(13):1774-1783.

6. Kim JY, Boyle L, Khashram M, Campbell D. Editor's Choice - Development and Validation of a Multivariable Prediction Model of Peri-operative Mortality in Vascular Surgery: The New Zealand Vascular Surgical Risk Tool (NZRISK-VASC). Eur J Vasc Endovasc Surg. 2021;61(4):657-663.

7. Feinglass J, Pearce WH, Martin GJ, Gibbs J, Cowper D, Sorensen M, et al. Postoperative and late survival outcomes after major amputation: findings from the Department of Veterans Affairs National Surgical Quality Improvement Program. Surgery. 2001;130(1):21-29.

8. Ambler GK, Thomas-Jones E, Edwards AGK, Twine CP. Prognostic Risk Modelling for Patients Undergoing Major Lower Limb Amputation: An Analysis of the UK National Vascular Registry. Eur J Vasc Endovasc Surg. 2020;59(4):606-613.

9. Tang TY, Prytherch DR, Walsh SR, Athanassoglou V, Seppi V, Sadat U, et al. The development of a VBHOM-based outcome model for lower limb amputation performed for critical ischaemia. Eur J Vasc Endovasc Surg. 2009;37(1):62-66.

10. Franchin M, Palermo V, Iannuzzi C, Rivolta N, Mozzetta G, Tozzi M, et al. A predictive score for 30-day survival for patients undergoing major lower limb amputation for peripheral arterial obstructive disease. Updates Surg. 2021;73(5):1989-2000.

11. Patterson AJ, Degnan AJ, Walsh SR, Eltayeb M, Scout EF, Clarke JM, et al. Efficacy of VBHOM to predict outcome following major lower limb amputation. Vasc Endovascular Surg. 2012;46(5):369-373.

12. Wied C, Foss NB, Kristensen MT, Holm G, Kallemose T, Troelsen A. Surgical apgar score predicts early complication in transfemoral amputees: Retrospective study of 170 major amputations. World J Orthop. 2016;7(12):832-838.

13. Norvell DC, Thompson ML, Boyko EJ, Landry G, Littman AJ, Henderson WG, et al. Mortality prediction following non-traumatic amputation of the lower extremity. Br J Surg. 2019;106(7):879-888.

14. Campbell D, Boyle L, Soakell-Ho M, Hider P, Wilson L, Koea J, et al. National risk prediction model for perioperative mortality in non-cardiac surgery. Br J Surg. 2019;106(11):1549-1557.

15. Czerniecki JM, Thompson ML, Littman AJ, Boyko EJ, Landry GJ, Henderson WG, et al. Predicting reamputation risk in patients undergoing lower extremity amputation due to the complications of peripheral artery disease and/or diabetes. Br J Surg. 2019;106(8):1026-1034.

16. Donati A, Ruzzi M, Adrario E, Pelaia P, Coluzzi F, Gabbanelli V, et al. A new and feasible model for predicting operative risk. Br J Anaesth. 2004;93(3):393-399.

17. Breitenstein S, DeOliveira ML, Raptis DA, Slankamenac K, Kambakamba P, Nerl J, et al. Novel and simple preoperative score predicting complications after liver resection in noncirrhotic patients. Ann Surg. 2010;252(5):726-734.

18. Simons JP, Ng SC, Hill JS, Shah SA, Zhou Z, Tseng JF. In-hospital mortality from liver resection for hepatocellular carcinoma: a simple risk score. Cancer. 2010;116(7):1733-1738.

19. Andres A, Toso C, Moldovan B, Schiffer E, Rubbia-Brandt L, Terraz S, et al. Complications of elective liver resections in a center with low mortality: a simple score to predict morbidity. Arch Surg. 2011;146(11):1246-1252.

20. Hill JS, Zhou Z, Simons JP, Ng SC, McDade TP, Whalen GF, et al. A simple risk score to predict in-hospital mortality after pancreatic resection for cancer. Ann Surg Oncol. 2010;17(7):1802-1807.

21. Venkat R, Puhan MA, Schulick RD, Cameron JL, Eckhauser FE, Choti MA, et al. Predicting the risk of perioperative mortality in patients undergoing pancreaticoduodenectomy: a novel scoring system. Arch Surg. 2011;146(11):1277-1284.

22. Greenblatt DY, Kelly KJ, Rajamanickam V, Wan Y, Hanson T, Rettammel R, et al. Preoperative factors predict perioperative morbidity and mortality after pancreaticoduodenectomy. Ann Surg Oncol. 2011;18(8):2126-2135.

23. Ragulin-Coyne E, Carroll JE, Smith JK, Witkowski ER, Ng SC, Shah SA, et al. Perioperative mortality after pancreatectomy: a risk score to aid decision-making. Surgery. 2012;152(3 Suppl 1):S120-127.

24. Uzunoglu FG, Reeh M, Vettorazzi E, Ruschke T, Hannah P, Nentwich MF, et al. Preoperative Pancreatic Resection (PREPARE) score: a prospective multicenter-based morbidity risk score. Ann Surg. 2014;260(5):857-863; discussion 863-854.

Supplementary Table 7: Supplementary References

| SR1 | Pettigrew, R. A. & Hill, G. L. Indicators of surgical risk and clinical judgement. Br J Surg 73, 47-51 (1986). <https://doi.org/10.1002/bjs.1800730121> |
| --- | --- |
| SR2 | Pettigrew, R. A., Burns, H. J. & Carter, D. C. Evaluating surgical risk: the importance of technical factors in determining outcome. Br J Surg 74, 791-794 (1987). <https://doi.org/10.1002/bjs.1800740912> |
| SR3 | Arvidsson, S., Ouchterlony, J., Sjostedt, L. & Svardsudd, K. Predicting postoperative adverse events. Clinical efficiency of four general classification systems. The project perioperative risk. Acta Anaesthesiol Scand 40, 783-791 (1996). <https://doi.org/10.1111/j.1399-6576.1996.tb04533.x> |
| SR4 | Pons, J. M. et al. Subjective versus statistical model assessment of mortality risk in open heart surgical procedures. Ann Thorac Surg 67, 635-640 (1999). <https://doi.org/10.1016/s0003-4975(98)01326-5> |
| SR5 | Avidan, M. S. et al. Comparison of structured use of routine laboratory tests or near-patient assessment with clinical judgement in the management of bleeding after cardiac surgery. Br J Anaesth 92, 178-186 (2004). <https://doi.org/10.1093/bja/aeh037> |
| SR6 | Markus, P. M. et al. Predicting postoperative morbidity by clinical assessment. Br J Surg 92, 101-106 (2005). <https://doi.org/10.1002/bjs.4608> |
| SR7 | Kaafarani, H. M. et al. Does surgeon frustration and satisfaction with the operation predict outcomes of open or laparoscopic inguinal hernia repair? J Am Coll Surg 200, 677-683 (2005). <https://doi.org/10.1016/j.jamcollsurg.2004.11.018> |
| SR8 | Hobson, S. A., Sutton, C. D., Garcea, G. & Thomas, W. M. Prospective comparison of POSSUM and P-POSSUM with clinical assessment of mortality following emergency surgery. Acta Anaesthesiol Scand 51, 94-100 (2007). <https://doi.org/10.1111/j.1399-6576.2006.01167.x> |
| SR9 | Woodfield, J. C., Pettigrew, R. A., Plank, L. D., Landmann, M. & van Rij, A. M. Accuracy of the surgeons' clinical prediction of perioperative complications using a visual analog scale. World J Surg 31, 1912-1920 (2007). <https://doi.org/10.1007/s00268-007-9178-0> |
| SR10 | Smith, D. D. & McCahill, L. E. Predicting life expectancy and symptom relief following surgery for advanced malignancy. Ann Surg Oncol 15, 3335-3341 (2008). <https://doi.org/10.1245/s10434-008-0162-1> |
| SR11 | Burgos, E. et al. Predictive value of six risk scores for outcome after surgical repair of hip fracture in elderly patients. Acta Anaesthesiol Scand 52, 125-131 (2008). <https://doi.org/10.1111/j.1399-6576.2007.01473.x> |
| SR12 | Karliczek, A. et al. Surgeons lack predictive accuracy for anastomotic leakage in gastrointestinal surgery. Int J Colorectal Dis 24, 569-576 (2009). <https://doi.org/10.1007/s00384-009-0658-6> |
| SR13 | Bakaeen, F. G. et al. Aortic valve replacement: mortality predictions of surgeons versus risk model. J Surg Res 163, 1-6 (2010). <https://doi.org/10.1016/j.jss.2010.03.015> |
| SR14 | Cornwell, L. D. et al. Predicting mortality in high-risk coronary artery bypass: surgeon versus risk model. J Surg Res 174, 185-191 (2012). <https://doi.org/10.1016/j.jss.2011.09.011> |
| SR15 | Jain, R., Duval, S. & Adabag, S. How accurate is the eyeball test?: a comparison of physician's subjective assessment versus statistical methods in estimating mortality risk after cardiac surgery. Circ Cardiovasc Qual Outcomes 7, 151-156 (2014). <https://doi.org/10.1161/CIRCOUTCOMES.113.000329> |
| SR16 | Glasgow, R. E. et al. Comparison of prospective risk estimates for postoperative complications: human vs computer model. J Am Coll Surg 218, 237-245 e231-234 (2014). <https://doi.org/10.1016/j.jamcollsurg.2013.10.027> |
| SR17 | Farges, O. et al. "Surgeons' intuition" versus "prognostic models": predicting the risk of liver resections. Ann Surg 260, 923-928; discussion 928-930 (2014). <https://doi.org/10.1097/SLA.0000000000000961> |
| SR18 | Promberger, R. et al. Can a surgeon predict the risk of postoperative hypoparathyroidism during thyroid surgery? A prospective study on self-assessment by experts. Am J Surg 208, 13-20 (2014). <https://doi.org/10.1016/j.amjsurg.2013.11.007> |
| SR19 | Ulyett, S. et al. Clinical assessment before hepatectomy identifies high-risk patients. J Surg Res 198, 87-92 (2015). <https://doi.org/10.1016/j.jss.2015.05.044> |
| SR20 | Sammour, T. et al. A simple web-based risk calculator ([www.anastomoticleak.com](http://www.anastomoticleak.com/)) is superior to the surgeon's estimate of anastomotic leak after colon cancer resection. Tech Coloproctol 21, 35-41 (2017). <https://doi.org/10.1007/s10151-016-1567-7> |
| SR21 | Woodfield, J. C. et al. Accuracy of the Surgeons' Clinical Prediction of Postoperative Major Complications Using a Visual Analog Scale. Med Decis Making 37, 101-112 (2017). <https://doi.org/10.1177/0272989X16651875> |
| SR22 | Samim, M. et al. Surgeons' assessment versus risk models for predicting complications of hepato-pancreato-biliary surgery (HPB-RISC): a multicenter prospective cohort study. HPB (Oxford) 20, 809-814 (2018). <https://doi.org/10.1016/j.hpb.2018.02.635> |
| SR23 | Kohler, J., Glass, N., Noiseux, N. O., Callaghan, J. J. & Miller, B. J. Might Doctors Really "Know Best"?: Utilizing Surgeon Intuition to Strengthen Preoperative Surgical Risk Assessment. Iowa Orthop J 38, 203-208 (2018). |
| SR24 | George, E. L. et al. Comparison of Surgeon Assessment to Frailty Measurement in Abdominal Aortic Aneurysm Repair. J Surg Res 248, 38-44 (2020). <https://doi.org/10.1016/j.jss.2019.11.005> |
| SR25 | Vanbrugghe, C. et al. Prospective study on predictability of complications by pancreatic surgeons. Langenbecks Arch Surg 405, 155-163 (2020). <https://doi.org/10.1007/s00423-020-01866-3> |
| SR26 | Zaruta, D. A. et al. Quantifying Surgeon Intuition Using a Judgment Analysis Model: Surgeon Accuracy of Predicting Patient-Reported Outcomes in Patients Undergoing Hip Arthroscopy for Femoroacetabular Impingement Is Moderate at Best. Arthrosc Sports Med Rehabil 5, e11-e19 (2023). <https://doi.org/10.1016/j.asmr.2022.09.010> |
| SR27 | Marwaha, J. S. et al. Quantifying the Prognostic Value of Preoperative Surgeon Intuition: Comparing Surgeon Intuition and Clinical Risk Prediction as Derived from the American College of Surgeons NSQIP Risk Calculator. J Am Coll Surg 236, 1093-1103 (2023). <https://doi.org/10.1097/XCS.0000000000000658> |
| SR28 | Wu, J. et al. The role of surgeon's intuition for acute type A aortic dissection in an era of evidence-based medicine: a prospective cohort study. J Thorac Dis 15, 5525-5533 (2023). <https://doi.org/10.21037/jtd-23-630> |
| SR29 | Gwilym, B. L. et al. Short-term risk prediction after major lower limb amputation: PERCEIVE study. Br J Surg 109, 1300-1311 (2022). <https://doi.org/10.1093/bjs/znac309> |
| SR30 | Gwilym, B. L. et al. Long-term risk prediction after major lower limb amputation: 1-year results of the PERCEIVE study. BJS Open 8 (2024). <https://doi.org/10.1093/bjsopen/zrad135> |
| SR31 | Berrigan, M. T. et al. Leveraging American Society of Anesthesiologists Physical Status Classification and Surgeon Risk Estimates to Stratify Surgical Risk: A Prospective Observational Study. J Surg Res 310, 323-330 (2025). <https://doi.org/10.1016/j.jss.2025.03.067> |
| SR32 | Kumar, R. M. et al. Are renal tumour scoring systems better than clinical judgement at predicting partial nephrectomy complexity? Can Urol Assoc J 11, 199-203 (2017). <https://doi.org/10.5489/cuaj.4228> |
| SR33 | Dyas, A. R. et al. Comparison of Preoperative Surgical Risk Estimated by Thoracic Surgeons vs a Standardized Surgical Risk Prediction Tool. Semin Thorac Cardiovasc Surg 34, 1378-1385 (2022). <https://doi.org/10.1053/j.semtcvs.2021.11.008> |
| SR34 | Ishikita, A. et al. Incremental value of machine learning for risk prediction in tetralogy of Fallot. Heart 110, 560-568 (2024). <https://doi.org/10.1136/heartjnl-2023-323296> |
| SR35 | El Moheb, M. et al. Artificial intelligence versus surgeon gestalt in predicting risk of emergency general surgery. J Trauma Acute Care Surg 95, 565-572 (2023). <https://doi.org/10.1097/TA.0000000000004030> |
